# twoddpcr: an R/Bioconductor package and Shiny app for Droplet Digital PCR analysis

**DOI:** 10.1093/bioinformatics/btx308

**Published:** 2017-05-05

**Authors:** Anthony Chiu, Mahmood Ayub, Caroline Dive, Ged Brady, Crispin J Miller

**Affiliations:** 1 Clinical and Experimental Pharmacology Group, Cancer Research UK Manchester Institute, The University of Manchester, Manchester, UK; 2 RNA Biology Group, Cancer Research UK Manchester Institute, The University of Manchester, Manchester, UK

## Abstract

**Summary:**

Droplet Digital PCR (ddPCR) is a sensitive platform used to quantify specific nucleic acid molecules amplified by polymerase chain reactions. Its sensitivity makes it particularly useful for the detection of rare mutant molecules, such as those present in a sample of circulating free tumour DNA obtained from cancer patients. ddPCR works by partitioning a sample into individual droplets for which the majority contain only zero or one target molecule. Each droplet then becomes a reaction chamber for PCR, which through the use of fluorochrome labelled probes allows the target molecules to be detected by measuring the fluorescence intensity of each droplet. The technology supports two channels, allowing, for example, mutant and wild type molecules to be detected simultaneously in the same sample. As yet, no open source software is available for the automatic gating of two channel ddPCR experiments in the case where the droplets can be grouped into four clusters. Here, we present an open source R package ‘twoddpcr’, which uses Poisson statistics to estimate the number of molecules in such two channel ddPCR data. Using the Shiny framework, an accompanying graphical user interface (GUI) is also included for the package, allowing users to adjust parameters and see the results in real-time.

**Availability and implementation:**

twoddpcr is available from Bioconductor (3.5) at https://bioconductor.org/packages/twoddpcr/. A Shiny-based GUI suitable for non-R users is available as a standalone application from within the package and also as a web application at http://shiny.cruk.manchester.ac.uk/twoddpcr/.

**Package maintainer:**

anthony.chiu@cruk.manchester.ac.uk

Bio-Rad’s QX100 and QX200 Droplet Digital PCR (ddPCR) are platforms for quantifying the number of copies of a given nucleic acid molecule in a sample. Starting material is mixed with the necessary reagents to perform PCR and then partitioned into ~20 000 droplets that each become individual reaction chambers for PCR.

By distributing the target sequence across ~20 000 droplets the platform gains in sensitivity, making it well-suited for tasks such as the detection of small amounts of tumour DNA against a larger background of wild-type DNA in circulating free DNA (cfDNA) extracted from patient blood (Hindson *et al.*, 2011). 

Conditions are set up on the assumption that target molecules are Poisson distributed across the droplets, and designed to maximize the number of droplets that each contain only a single copy. The mixture is then thermocycled, resulting in amplification. TaqMan reagents are included in the reaction mixture and upon annealing of the TaqMan probe to the PCR product, the quenching component of the probe is removed by Taq polymerase, resulting in an increase in fluorescence. The number of droplets that fluoresce can then be used to estimate the concentration and allelic fraction of the target molecule in the sample. The Bio-Rad platform supports two channels, allowing different fluorochromes to be used to detect two different target sequences in the sample at once.

The goal of data analysis is therefore to use the two channel fluorescence data to classify droplets into four sets: positive in both channels (PP), positive only in Channel 1 (PN), positive only in Channel 2 (NP), or negative in both channels (NN). Having established the number of positive droplets in each channel, Poisson statistics can then be used to estimate the starting concentrations and hence the absolute copy numbers.

Droplets can vary in brightness. This is a particular problem for cfDNA samples such as in (Wang *et al.*, 2016; Zhu *et al.*, 2015), where the droplet amplitude plots may be noisy and the PN cluster can ‘lean’ and the NP cluster can ‘lift’. In a study by (Whale *et al.*, 2016), the reasons for these phenomena may be due to the cross-hybridization of probes and the presence of multiple target molecules in the same droplet. Therefore, classifying droplets on absolute fluorescence intensity can be problematic, resulting in a number of alternative gating strategies being proposed.

For example, the Javascript-based web application ‘definetherain’ uses *k*-means clustering to classify droplets in one channel data only (Jones *et al.*, 2014). It removes droplets between the two clusters that are more than three times the standard deviation away from their respective cluster means. These droplets are colloquially known as ‘rain’.

The ‘ddpcRquant’ R package estimates the parameters for a generalized extreme value distribution, and uses 0.995 percentiles to set a threshold for one channel data (Trypsteen *et al.*, 2015). This is repeated 100 times and the mean threshold taken as the final cut-off. A modified version of this approach is included in the R package ‘dpcR’, which is a toolset for analysing data from various digital PCR platforms (Burdukiewicz *et al.*, 2017).

Another R package, ‘ddpcr’, gates droplets in a specific class of experiments, where the droplets cluster into three distinct classes: NN, PP, and either NP or PN, but not both. The package also supports manual gating of more general two channel experiments (Attali *et al.*, 2016).

Here, we present ‘twoddpcr’, an open source package for the R programming language (R Core Team, 2016) designed for gating general two channel ddPCR experiments, allowing for the integration of ddPCR analyses of such experiments into bioinformatics pipelines. Unlike existing packages, it supports manual and automatic gating of experiments where droplets fall into all four classes (NN, NP, PN and PP), making it suitable for the detection of mutant molecules in challenging samples such as cfDNA. In addition, the ‘twoddpcr’ package adopts a novel approach for further refining such classifications; this approach uses Mahalanobis distance and will be discussed shortly. An accompanying web application built using the Shiny framework (Chang *et al.*, 2017) provides a graphical user interface (GUI), enabling non-R users to use the package and means that parameters can be adjusted easily in order to view the results in real-time. The package accepts raw data in the form of droplet amplitude files exported from Bio-Rad’s QuantaSoft.

Software is provided as a Bioconductor (Huber *et al.*, 2015) package ‘twoddpcr’, making particular use of the ‘class’ (Venables and Ripley, 2002) and ‘stats’ packages for classifying droplets. The package revolves around the use of ddpcrPlate S4 objects, which are extended from the SimpleList S4 class from the ‘S4Vectors’ package (Pagès *et al.*, 2017). A number of other R packages were used for visualization purposes (Carr *et al.*, 2016; Neuwirth, 2014; Wickham, 2009). The Shiny-based GUI is included in the package as a standalone application and is also available at http://shiny.cruk.manchester.ac.uk/twoddpcr/.

The package uses *k*-means clustering to classify droplets by identifying four candidate groups. This approach is particularly effective when these groups are not separated into four non-overlapping quadrants; an example of this can be seen in Figure 1.

**Fig. 1:**
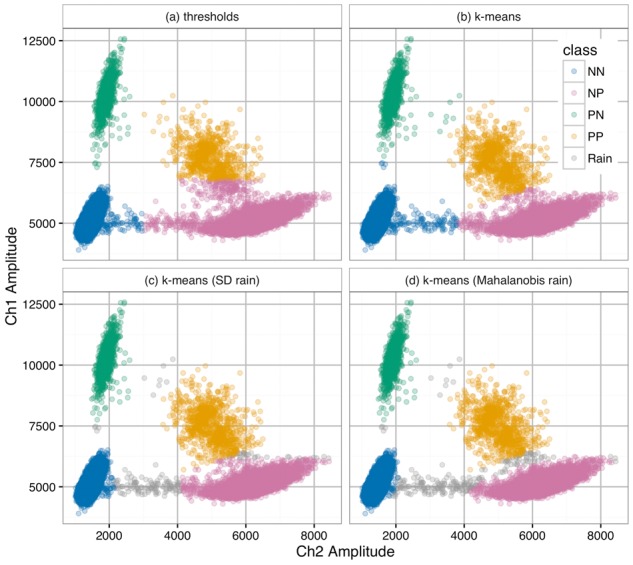
Various classifications of the droplet amplitudes from the KRASdata dataset. (**a**) Setting thresholds with the Channel 1 threshold at 6789 and Channel 2 threshold at 3000. (**b**) The *k*-means classification. (**c**) The *k*-means classification with rain defined by standard deviations of clusters. (**d**) The *k*-means classification with rain defined by Mahalanobis distance

In addition, the package provides a Mahalanobis distance-based approach to refine the classifications by defining ellipses around each candidate group, taking their rotations into account. For each cluster, *c*, the mean ${\vec{\mu }}_{c}$ and covariance Σ_*c*_ is calculated. This allows the squared Mahalanobis distance (Mahalanobis, 1936) between a point $\vec{x}$ and a distribution *M_c_* that has mean ${\vec{\mu }}_{c}$ and covariance Σ_*c*_ to be calculated by $d_{M_{c}}(\vec{x})={\left(\vec{x}-{\vec{\mu }}_{c}\right)}^{T}\Sigma_{c}(\vec{x}-{\vec{\mu }}_{c})$. A maximum distance *m_c_* can be fixed by the user so that all droplets $\vec{x}$ where $d_{M_{c}}(\vec{x})\leq m_{c}$ will be considered as part of the class; remaining droplets will be treated as ‘rain’. The *k*-nearest neighbour (*k*-NN) algorithm is also provided as an option.

Once the number of positive and negative droplets in each channel has been determined, the actual number of fragments in each sample can be estimated. The package assumes that the molecules of both targets are Poisson distributed: Suppose that the volume of each droplet is *V* (0.85nl by default), and that there are *P_T_* droplets containing the amplified target type *T*, and *R* total accepted droplets in the well. The concentration of targets per microlitre can be estimated by $-\text{log}\;\left(1-P_{T}/R\right)/V$, which is derived from the probability mass function of the Poisson distribution.

We illustrate the use of the package using sample data as follows: A549 KRAS mutant cell lines and H1048 KRAS wild type cells were used to provide triplicate samples with 5% mutant DNA at increasing amounts of starting material (at 1 ng, 4 ng, 16 ng, 64 ng). This dataset is included as KRASdata in the package.

Example data are shown in Figure 1a, using independent linear gating on each channel. With these data it is not possible to choose a threshold that correctly defines the NP and PP clusters. While *k*-means classification performs better, as shown in Figure 1b, the lack of separation between clusters NP and PP make it difficult to accurately define ambiguous droplets (‘rain’) that lie between the main clusters.

As with the (Jones *et al.*, 2014) and (Attali *et al.*, 2016) publications, twoddpcr’s sdRain method identifies droplets that lie further than *n* (by default 5) multiples of the standard deviation from the mean of each cluster. This leads to improved classification but since it still relies on linear gates, separation between NP and PP clusters is still unsatisfactory (Fig. 1c).

In contrast, the Mahalanobis-based mahalanobisRain refinement better separates the groups because it takes the cluster rotations into account (Fig. 1d). This method requires that the parameters are manually adjusted. This ensures that the ellipses are large enough to include most droplets of each class but small enough to remove ambiguous droplets.

In conclusion, twoddpcr uniquely provides open source software in R and Bioconductor for the analysis of two channel ddPCR data. The included Shiny app provides a GUI that allows for parameters in the analysis to be adjusted and visualized in real-time.
